# Multicenter Renal Pharmacist Group—Pharmaceutical Care for Patients with Renal Impairment at Four Non-University Hospitals in Germany

**DOI:** 10.3390/jcm14134530

**Published:** 2025-06-26

**Authors:** Sarah Seiberth, Katrin Bayerlein, Ann-Kristin Gerke, Angela Ihbe-Heffinger, Hans-Paul Schobel, Jana Rudolph, Sarah Leuschner, Philipp Müller, Ina Richling, Boris Owandner, Tanja Schmidt-Schnaubelt, Meike Sieg, Larissa Albus, Andreas von Ameln-Mayerhofer, Dorothea Strobach

**Affiliations:** 1 Doctoral Program Clinical Pharmacy, University Hospital of the Ludwig-Maximilians-University, 81377 Munich, Germany; 2 Hospital Pharmacy and Division of Nephrology, Starnberg Hospital, Academic Teaching Hospital of LMU, 82319 Starnberg, Germany; 3 Hospital Pharmacy, Rudolf Virchow Hospital Glauchau, Academic Teaching Hospital of the University Hospital, 08371 Glauchau, Germany; 4 Hospital Pharmacy and Department of Surgery, Katholische Kliniken im Märkischen Kreis, 58638 Iserlohn, Germany; 5 Hospital Pharmacy, Hospital Sindelfingen-Boeblingen, 71065 Sindelfingen, Germany

**Keywords:** renal impairment, renal pharmacist, drug-related problems, medication-related problems, pharmacist-led medication review, pharmaceutical care

## Abstract

**Background:** The project ‘Multicenter Renal Pharmacist Group—Implementation of Pharmaceutical Care for Patients with Renal Impairment at four Non-University Hospitals in Germany’ started in the beginning of 2020 with the goal to establish high-quality pharmaceutical care to improve patient safety for hospitalized patients with renal impairment at German non-university hospitals. Pharmaceutical service quality should be optimized by intense and effective intraprofessional collaboration within the network. **Methods:** Over a period of two years (2020–2022), we implemented renal pharmacists (RPs) for patients with renal impairment (RI) at four non-university hospitals in Germany (Starnberg Hospital, Rudolf Virchow Hospital Glauchau, Catholic Hospital in the Märkisch District (KKiMK), and Hospital Sindelfingen-Boeblingen). The RPs conducted medication analyses identifying renal-drug-related problems (rDRPs) two to five days a week. The rDRPs, including recommendations to solve them, were forwarded to the attending physicians via written consultations or personally during ward rounds. The RPs were mentored by a renal pharmacist expert from LMU Munich and formed a multicentered team with close collaboration. Data about the RP service were collected and were retrospectively evaluated. **Results:** During the two-year project period, a total of 3924 patients from various disciplines were visited across all four locations. In total, 1425 patients (36.3%; with a range from 22.7 to 56.4% between hospitals) received one or more interventions by RPs concerning 2454 rDRPs (a median of one to three rDRPs per patient). In cooperation with the physicians, 77.6 to 88.2% of the rDRPs were solved. The most common causes were ‘dosage too high’ and ‘contraindication’. **Conclusion:** The implementation of pharmaceutical care for patients with renal impairment at four non-university hospitals in Germany increased appropriate prescribing by physicians. The multicenter team proved to be an excellent support for the newly established services.

## 1. Introduction

Renal impairment (RI) is a major health issue concerning about 10% of the adult population according to an evaluation including 11 western countries [1]. The consequences are severe with frequent hospital admissions, a higher mortality rate, and higher costs of care [1]. In Germany, RI has been described in 2–8% of the population aged 18–79 years, rising to 20–25% in patients older than 70 years [2,3]. Importantly, only 25% of the patients knew about their organ impairment and only two-thirds of these received medical surveillance for this reason [2]. RI is found in 20–50% of hospitalized patients, depending on the medical specialty [4,5,6,7]. This includes patients with known RI, but also patients with previously unknown renal disease or acute worsening of kidney function.

RI is a well-known risk factor for drug safety; however, studies in the ambulatory as well as hospital setting have found inappropriate prescribing in up to 80% of patients [8]. This has been linked to a higher rate of adverse drug reactions, longer hospital stay, and higher mortality [8,9]. Renal risk drugs either show altered pharmacokinetics or pharmacodynamics in RI or directly affect renal function [4,10]. Drug-related problems (DRPs) in RI, most often concerning inappropriate dose adjustment or drug choice and contraindications, are frequent. For instance, a study in urologic patients of a university hospital found that more than 50% of the patients were taking renal risk drugs and more than 40% had renal DRPs [5].

Studies in different settings have evaluated how prescribing in RI can be improved. The interprofessional work of pharmacists and physicians was proven to be the most effective way, especially when direct feedback from pharmacists was given during prescribing [8]. In contrast, simply displaying the glomerular filtration rate (eGFR) was not effective [8]. Even in the setting of physician order entry systems with clinical decision support (CPOE-CDSS), which are supposed to increase appropriate prescribing, a pharmacist identified renal-drug-related problems in 11% of patients with RI [6]. Renal pharmacists, who focus on correct prescribing in hospitalized patients with RI, have been shown to reduce the length of hospital stay and to delay the progression of kidney disease [11,12]. Unfortunately, trained renal pharmacists will not always be available, especially in smaller, non-university hospitals. However, focus on patients with RI is important taking into account pharmacists’ time as a valuable resource and the expected clinical impact. To address these issues, a multicenter network was founded, including pharmacists of four non-university hospitals and an experienced university renal pharmacist, aiming to provide a framework for education as well as professional discussion and support. This study aimed to evaluate the renal pharmacists’ projects regarding chosen patient groups, detected renal DRPs, pharmacists’ interventions, and their acceptance rates. The overall aim is to study how renal pharmacists could be implemented more broadly in standard care to increase patient safety.

## 2. Materials and Methods

Since 2020, we have dispatched renal pharmacists (RPs) for patients with renal impairment (RI) at four non-university hospitals in Germany (Starnberg Hospital, Rudolf Virchow Hospital Glauchau, Catholic Hospital in the Märkisch District (KKiMK), and Hospital Sindelfingen-Boeblingen). Each hospital was granted funding for an RP position equal to 50% of a full-time pharmacist position over a period of two years. Renal pharmacist projects tailored to the four non-university hospitals’ needs were outlined and implemented in practice. Details about each hospital setting, frequency of medication analysis, and patients’ inclusion criteria are shown in Table 1. The included disciplines varied at each hospital, but all hospitals included the department of geriatrics and trauma surgery. The included patients, who received medication analysis by RPs, were selected automatically (except for one hospital) according to the glomerular filtration rate (eGFR). Most of the hospitals included patients with an eGFR smaller than 60 mL/min/1.73 m^2^. Data about their renal function are usually obtained at admission and then mostly daily during their hospital stay. Pharmacists always checked the latest eGFR for medication review and took into account additional calculations of renal function, like eGFR adjusted to the patients’ body surface area (mL/min) and by Cockroft and Gault formula (mL/min).

Focus of each project was the pharmaceutical care for patients with renal impairment, including medication analysis identifying renal-drug-related problems (rDRPs), defined as any DRP in relation to the renal function of patients. This would include dose adjustments to renal function, contraindications, the choice of drug regarding renal function, or the choice to stop a drug because of renal function. The renal pharmacists were conducting medication analyses, identifying rDRPs two to five days a week. They were working closely with the attending physicians, informing them about patients’ rDRPs and recommending interventions, solving them via written consultations or personally during rounds. Standard sources used by renal pharmacists to evaluate the drug therapy were the German Summary of Product Characteristics, the database Dosing.de (University of Heidelberg, Germany), and the Renal Drug Handbook (Editors: C. Ashley, A. Dunleavy; CRC Press, London, UK). The projects were supported by a renal pharmacist of the University Hospital LMU Munich, where this service had already been implemented.

Prior to the project start, the newly appointed renal pharmacists completed the online course ‘Fundamentals of Renal Therapeutics‘ from the Centre for Pharmacy Postgraduate Education (CPPE) at the University of Manchester [13]. The course teaches the main information about renal function and adequate medications, acute kidney injury, chronic kidney diseases, and dialysis. To complete the different modules, a quiz had to be passed at the end.

To support and benefit from each other, the renal pharmacists formed a multicentered team with a close collaboration mentored by a renal pharmacist expert from LMU Munich. They developed medication review processes for patients with renal impairment tailored to the need of each setting and initiated monthly online meetings to discuss complex patient cases, share their expertise concerning critical drugs, and improve problem solving skills for consultation with healthcare professionals.

### 2.1. Data Collection and Assessment

Pharmaceutical care activities of the RPs were documented and retrospectively evaluated. The number, sex, age, eGFR by CKD-EPI equation [mL/min/1.73 m^2^], weight, height, and number of drugs taken by patients who received a pharmacist-led medication analysis were recorded. For the documentation of the rDRP, the RPs chose their system individually and either the PCNE-classification, the German DokuPik system or classification following a former study were used [14,15,16]. Each hospital was responsible for its data collection. Details on the documentation were discussed and agreed on in the renal pharmacists’ group during several online meetings, and data were checked for plausibility by the renal pharmacist expert from LMU Munich.

### 2.2. Pharmacist’s Intervention

The renal pharmacist identified the rDRPs during medication analyses of the included patients. Details about each hospital are shown in Table 1. To identify the benefit of recruitment of a renal pharmacist, we retrospectively evaluated the change in prescriptions after renal pharmacist’s interventions. The acceptance of interventions was documented by each hospital separately by reviewing patients’ records.

### 2.3. Ethical Approval

The study was conducted in accordance with the principles of the Declaration of Helsinki. Because of the retrospective design, patient informed consent was not requested and not obtained, in accordance with the applicable statutory provisions under the Bavarian State Hospital Act (Art. 27 Para. 4 BayKrG). All data were anonymously recorded during clinical routine without any patients identifying information. Therefore, the local ethics committees at study sites waived the need for ethical approval.

### 2.4. Statistical Analysis

We used descriptive statistics to characterize patient population. Qualitative variables are presented with their frequency distribution. Quantitative variables are expressed as mean and standard deviation (SD) (normal distribution) or as median and interquartile range (without normal distribution). Data analyses and figures were completed using Microsoft Excel^®^ 2024 (Seattle, WA, USA).

## 3. Results

During the two-year project period, in total, 3924 patients received a pharmacist-led medication review (during chart rounds) by a renal pharmacist at all four hospitals. The demographic data for the different hospitals are shown in Table 2. In each hospital, the main patient population was female and geriatric with an age ≥ 75 years. In sum, 1425 patients (36.3%) were identified with renal-drug-related problems (a frequency between 22.7 and 56.4%, depending on the hospital). These patients took a median of 10 to 13 drugs, which the renal pharmacists reviewed for rDRPs.

**Figure 1:**
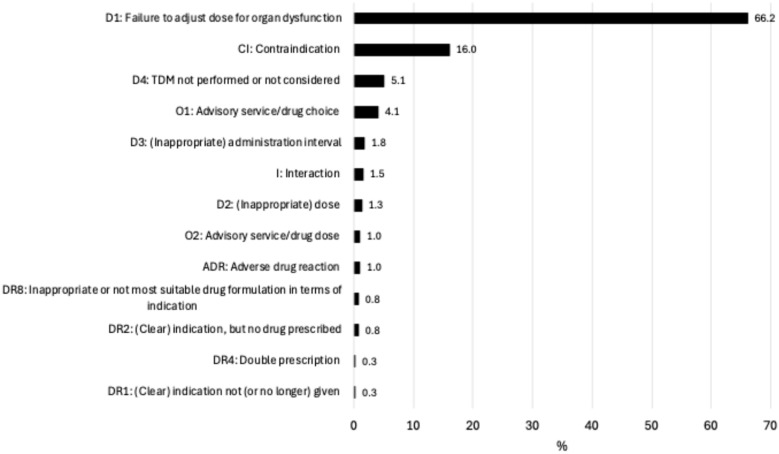
Doku Pik classification—type and frequency of rDRPs at Starnberg Hospital (n = 393).

**Figure 2:**
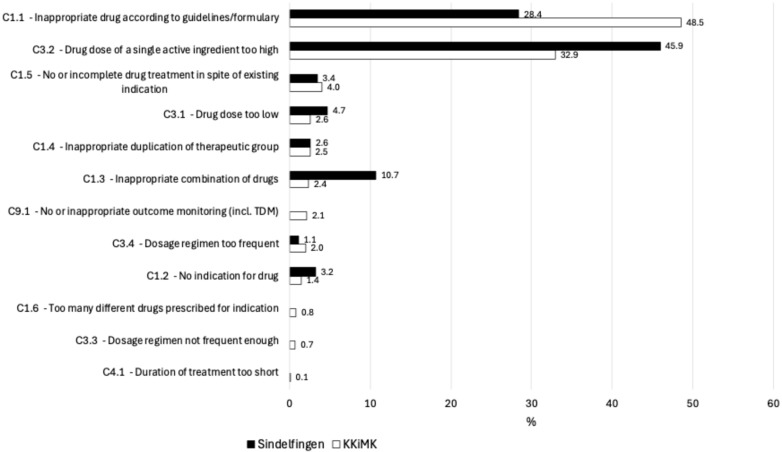
PCNE classification—type and frequency of rDRPs at KKiMK (n = 1275) vs. Sindelfingen Hospital (n = 468).

**Figure 3:**
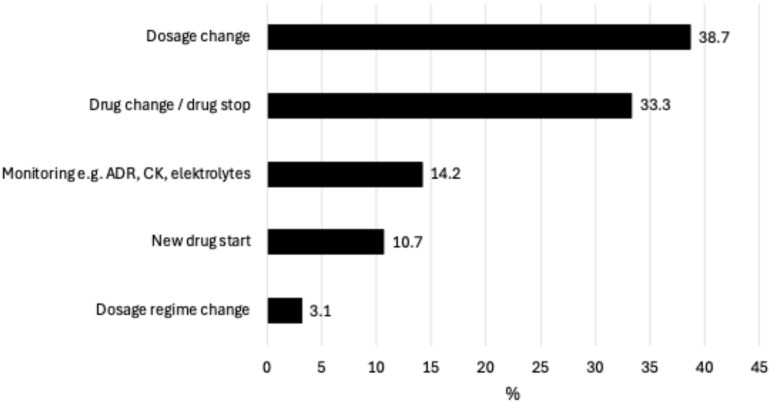
Type and frequency of interventions that were proposed from renal pharmacists to prescribers to solve rDRPs at Glauchau Hospital (n = 318). **ADR**: adverse drug reaction, **CK**: creatine kinase.

The pharmacists identified in total 2454 rDRPs leading to intervention proposals (Table 3), with a median of one to three rDRPs per patient. Figure 1 shows the rDRPs documented as Doku Pik (Starnberg hospital), Figure 2 shows those classified as PCNE (Catholic Hospital in the Märkisch District (KKiMK) vs. Hospital Sindelfingen-Boeblingen), and Figure 3 shows the interventions that were proposed from the renal pharmacist to the prescriber in a classification used in a former study (Rudolf Virchow Hospital Glauchau) [5]. The RPs identified ‘drug dose too high’ (32.9–66.2%) and ‘contraindication’ (16.0–48.5%) as the main causes of rDRPs at all locations.

After the renal pharmacists’ intervention, in cooperation with the physicians, between 77.6 and 88.2% of the rDRPs were solved, leading to a change in prescription.

**Figure 4:**
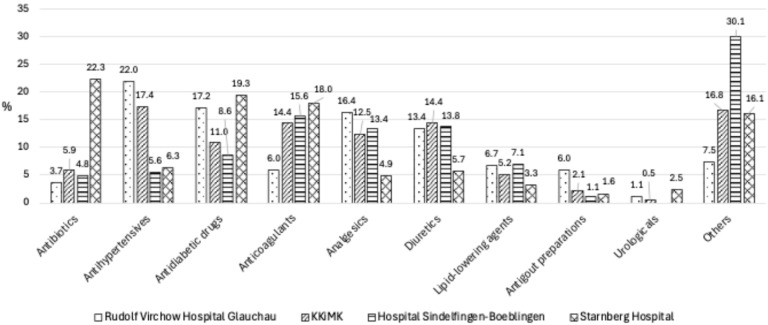
Type and frequency of drug classes that were found with rDRPs, comparing all four hospitals.

The main drug classes that have been identified to show rDRPs varied among hospitals and included antibiotics (3.7–22.3%), antihypertensives (5.6–22.0%), antidiabetic drugs (8.6–19.3%), anticoagulants (6.0–18.0%), analgesics (4.9–16.4%), diuretics (5.7–14.4%), lipid-lowering agents (3.3–7.1%), antigout preparations (1.1–6.0%), urologicals (0–2.5%), and others (7.5–30.1%) (Figure 4).

**Figure 5:**
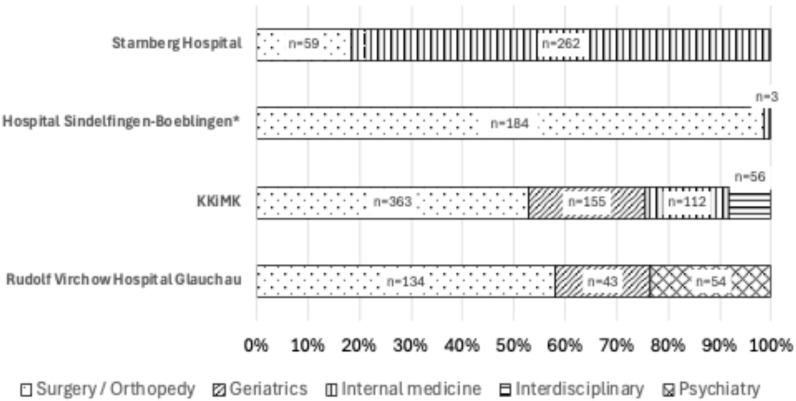
Distribution of disciplines from patients with renal-drug-related problems (rDRPs) (n = 1425). * Geriatric patients are included in surgery (Center of Geriatric Traumatology; all patients included in geriatric programs).

Figure 5 shows the distribution of the departments of patients showing rDRPs. In Starnberg Hospital, 81.6% of patients with rDRPs belonged to the Internal Medicine Department. On the other hand, at the other three hospitals, the patients with rDRPs were mainly from the departments of surgery and geriatrics (Hospital Sindelfingen-Boeblingen: 98.4%; KKiMK: 75.5%; and Rudolf Virchow Hospital Glauchau: 76.6%).

## 4. Discussion

Renal impairment is a well-known risk factor leading to renal-drug-related problems (rDRPs) and is found in 20–50% of hospitalized patients, depending on the medical specialty [4,5,6,7]. In addition, the interprofessional work of pharmacists and physicians is known to be the most effective way to solve rDRPs, especially when direct feedback from pharmacists is given when prescribing [8]. However, especially in smaller, non-university hospitals, trained renal pharmacists are not available due to the lack of data in these settings, organizational obstacles, and cost considerations. This multicenter project shows that renal pharmacists in non-university hospital settings can effectively foster patient safety by identifying rDRPs and working closely together with the attending physicians. In projects tailored to the needs of each hospital, rDRPs were found for 23% to 56% of the included patients. The higher rates were found in hospitals which included mostly or only patients from the departments of trauma surgery and geriatrics, reflecting the special need for additional pharmaceutical support in these departments. The recommendations of the RPs were deemed clinically relevant, as up to 88% of the prescriptions showing rDRPs were changed.

The successful implementation of new clinical pharmacy services is a demanding task requiring, e.g., educational and organizational issues. In this project, a multicenter renal pharmacist team was established, representing a novelty for German standards for hospital pharmacies. Newly appointed renal pharmacists (RPs) in four hospital pharmacies mentored by a renal pharmacist expert from a university hospital formed a team. The group developed medication review processes for patients with renal impairment tailored to the need of each setting and initiated monthly online meetings to discuss complex patient cases, share expertise concerning critical drugs, and improve problem solving skills for consultations with healthcare professionals.

In a multicenter project, the RPs collected essential data to demonstrate the importance and benefits of renal pharmacists in German non-university hospitals, taking on professional responsibility and providing their drug-oriented expertise. The multiprofessional collaboration of the RPs with the physicians has also intensified at each hospital. At the end of the project period, two hospital pharmacies had already been granted the financial continuation of an RP.

When a patient is admitted to hospital, responsibility for all drug therapy is transferred to the attending ward physician. However, it is difficult in terms of time and personnel to thoroughly consider every patient in all aspects of their drug therapy. In particular, patients with renal impairment often have rDRPs with their admission medication [4,5,8,17,18,19]. In three out of four of the project hospitals, the RPs mainly looked after patients in the surgical department, where the physician’s primary focus is on the surgical procedure. However, the clinical outcome on wards can be heavily influenced by drug therapy and existing or newly occurring rDRPs. In the project hospitals, the rate of the occurrence of rDRPs was between 26 and 47% in the reviewed patients. In comparison, in the hospital with mainly patients from the internal medical department, the RPs found one or more rDRPs in 23% of the reviewed patients.

The interprofessional work of pharmacists and physicians proved to be the most effective way, especially when direct feedback from pharmacists was given when prescribing [8]. A recent paper underlined that personal interaction, in addition to paper or digital reports, could help to improve the acceptance of recommendations made by RPs [16]. At all locations, the RPs informed the physicians about rDRPs during rounds or face-to-face, so that clinical questions could be discussed by the interprofessional team. Additionally, the RP gave the physicians a written consultation about a patient’s rDRP. One reason was the documentation; the other reason was that physicians could easily include the consultation in the discharge letter so that the transfer of information to the outpatient sector could continue. The inappropriate transfer of information from the hospital to the ambulatory setting regarding medication changes is a well-known risk for drug therapy safety [20]. Two of the hospitals even introduced a written pharmaceutical consultation in their electronic hospital information system to be able to directly transfer the information electronically in the discharge letter. This process made it possible to ensure the information flow to the outpatient sector.

Personal interaction between pharmacists and physicians promotes the acceptance of pharmaceutical recommendations [8], as well as encourages sustainable interprofessional learning for physicians and pharmacists. We see this finding in our project as well: the highest change in prescriptions is shown in the project in which the renal pharmacist cooperated closely with a nephrologist and made recommendations as an interprofessional team.

The aim of all RP projects was to support the ward physician in their daily work and improve the interface between outpatients and inpatients. The RPs not only analyzed the medications at admission, but they could also quickly address fluctuating kidney function by performing pharmacist-led medication reconciliation several times a week.

In order to save resources, it was important to select patients with impaired renal function in a time-saving manner. Three out of four projects developed processes to automatically filter patients with an eGFR under a specific value via the laboratory program. These projects were able to see 2 to 3.5 times the number of patients than the project that selected patients manually during the project period. Automatic selection versus manual selection from the lab software was faster and more effective as well as resource-saving. We recommend this approach for all further projects.

In comparison to previous studies, we found similar drug classes involved in rDRPs at all four project sites [4,5,6,8,10,16]. At the one location with mostly internal medicine patients, the drug class with the highest rate of DRPs was antibiotics, followed by antidiabetic drugs and anticoagulants. At the three other sites, which mainly worked with surgery patients, the most common drug classes with rDRPs were analgesics, anticoagulants, antihypertensives, diuretics, and antidiabetic drugs.

As a limitation of this project evaluation, we were not able to assess clinical data supporting the beneficial effect of the RPs as recommended [21]. This would have required a prospective or more complex retrospective data analysis, which was not within the time scope of the financial support granted for the project. However, the number of DRPs identified per patient and the high implementation rate of pharmacists’ recommendations underline the clinical value of the service. Also, it would have been interesting to go into more detail, for example, to study whether patient-specific factors, like age or number of drugs, were associated with a higher likelihood of rDRPs and in what circumstances the rate of acceptance of suggestions to alter the drug therapy was highest. These questions should be addressed in future studies. Additional questions for future evaluations include the time spent per patient by the renal pharmacist, which was not documented in our project. This is closely linked to the additional interesting question of the cost of the renal pharmacists versus the economic impact of the solved rDRPs, which was also beyond the scope of this project. An additional limitation are the variations in documentation between the four project sites, which, on reflection, should have been agreed upon at the start of the study but could not be harmonized later. We think the results would have been even more convincing if one documentation system was used. However, the benefit of the renal pharmacist was shown in all hospitals regardless of the used documentation system. However, we do not consider the selection of different patient groups and wards as a limitation but as a strength, reflecting individual clinical needs at the project sites. This emphasizes the broad need of an RP as part of a clinical pharmacy service across hospitals with different characteristics.

The project was extremely successful for two reasons: First, this individualized approach was tailored to the participating hospitals regarding the chosen wards, choice of patient group, and how the renal pharmacist interacted within the therapeutic team. Second, a mandatory advanced certified online course for the participating pharmacists prior to the project’s start and the close professional exchange in regular group meetings ensured the high quality of the renal pharmacists’ work from the beginning. The renal pharmacist group embodies what great teamwork should look like and how it can be applied effectively to improve patient care.

For the future recruitment of renal pharmacists across hospitals in Germany or other countries, important recommendations can be suggested following this study. Projects should be tailored to the hospital’s needs depending on the medical specialties and patient groups cared for. To be successful, an interprofessional approach with the close cooperation of pharmacists and physicians is of great importance, and face-to-face communication is recommended. Pharmacists should be able to screen for patients with RI by automated eGFR alerts to realize the most time-efficient way to conduct medication reviews. Finally, pharmacists should be trained appropriately in all aspects of renal impairment and drug therapy before starting to work as an RP and seek continuous learning. Establishing a nationwide expert group of renal pharmacists and physicians interested in the field would help to increase awareness and appropriate drug therapy for patients with renal impairment.

## 5. Conclusions

The implementation of pharmaceutical care for patients with renal impairment at four non-university hospitals in Germany increased appropriate prescribing by physicians and was perpetuated after the project period to foster patient safety. Pharmaceutical service quality was optimized by intense and effective interprofessional collaboration within the network. The project’s success is based on the close interprofessional cooperation between the physicians and pharmacists to ensure patient safety.

## Figures and Tables

**Table 1:** Basic parameters of the four renal pharmacist projects. All locations used paper charts. **eGFR**: glomerular filtration rate, **CKD-EPI**: Chronic Kidney Disease Epidemiology Collaboration, **RI**: renal impairment.

Pharmacy	Rudolf Virchow Hospital Glauchau	Catholic Hospital in the Märkisch District (KKiMK)	Hospital Sindelfingen-Boeblingen	Starnberg Hospital *
**Hospital characteristics**	320-bed hospital for basic and standard care	408-bed hospital for basic and standard care	434-bed hospital for basic and standard care	312-bed hospital for basic and standard care
**Project period**	02/2020–01/2022	02/2020–01/2022	06/2020–05/2022	01/2020–12/2021
**Included department**	Geriatrics; Psychiatry; General, Visceral, and Vascular Surgery; Orthopedy; and Trauma Surgery	Geriatrics, Trauma Surgery	Geriatrics, Trauma Surgery	All departments
**Initially included patients** **eGFR by CKD-EPI** **[mL/min/1.73 m^2^]**	<60	≤50	<60	<30 since 07/2020 <45 (Dep. of Surgery)
**Selection of patients with RI**	Automatically selected via LabCentre ^a^	Automatically selected via MOMO ^b^ in ORBIS ^c^	Manually selected from lab software	Automatically selected via ORBIS ^c^
**Medication analysis**	Every weekday; plus once weekly during ward round	3 times per week	2–3 times per week; plus once weekly during ward round	2 times per week
**Interventions**	Written consultation in paper chart and face-to-face/by phone	Face-to-face and written consultation in ORBIS	Face-to-face, otherwise written consultation in paper chart	By phone/face-to-face and written consultation in Orbis (as of 11/2021)

* Medication analysis in cooperation with Prof. Dr. Hans-Paul Schobel of the Department of Nephrology. ^a^ LabCentre is a lab software from Mesalvo GmbH. ^b^ MOMO is a medical controlling program from Tipli GmbH. ^c^ ORBIS is a clinical workplace system from Dedalus GmbH.

**Table 2:** Demographic data of all patients who received medication reviews (during chart rounds) by a renal pharmacist. Data are quoted as the mean ± SD, the median (interquartile range), or n (%). **BSA**: body surface area, **BMI**: body mass index.

	Rudolf Virchow Hospital Glauchau	KKiMK	Hospital Sindelfingen-Boeblingen	Starnberg Hospital
**Total**	894	1217	396	1417
**Females**	560 (62.6)	801 (65.8)	246 (62.1)	789 (55.7)
**Age** [years]	79 (48–99)	83 (33–101)	83 (31–99)	83 (45–101)
18–39	0 (0.0)	1 (0.1)	1 (0.3)	0 (0.0)
40–59	33 (3.7)	27 (2.2)	9 (2.3)	25 (1.8)
60–64	31 (3.4)	23 (1.9)	3 (0.8)	30 (2.1)
65–74	174 (19.5)	153 (12.6)	46 (11.6)	149 (10.5)
≥75	656 (73.4)	1013 (83.2)	338 (85.1)	1213 (85.6)
**BSA [m^2^]**	1.88 ± 0.2 (n = 645)	1.86 ± 0.2 (n = 1015)	1.81 ± 0.24 (n = 228)	1.86 ± 0.2 (n = 841)
**BMI** [kg/m^2^]	26.9 (13.8–49.9) (n = 645)	27.0 (16.0–70.3) (n = 1015)	25.6 (13.8–62.5) (n = 228)	25.5 (12.4–54.4) (n = 841)
**Renal impairment**				
**<15**	12 (1.3)	81 (6.6)	20 (5.1)	131 (9.1)
**15–29**	126 (14.1)	355 (29.2)	77 (19.4)	659 (45.9)
**30–44**	271 (30.3)	537 (44.1)	106 (26.8)	541 (37.7)
**45–59**	485 (54.3)	244 (20.1)	193 (48.7)	86 (6.0) *
[mL/min/1.73 m^2^]				
**No. of drugs ^#^**	9106	12,139	4586	14,624
per patient	10 (1–25)	10 (1–24)	12 (3–22)	10 (0–22)

^#^ Medications on demand are not included; * Patients ≥ 45 mL/min/1.73 m^2^ are included by special request of ward physicians.

**Table 3:** Patients with renal-drug-related problems. Data are quoted as the mean ± SD, the median (interquartile range), or n (%). **BSA**: body surface area, **BMI**: body mass index.

	Rudolf Virchow Hospital Glauchau	KKiMK	Hospital Sindelfingen-Boeblingen	Starnberg Hospital
**Total**	231 (25.8)	686 (56.4)	187 (47.2)	321 (22.7)
**Females**	151 (65.4)	451 (65.7)	109 (58.3)	176 (54.8)
**Age** [years]	81 (54–96)	83 (41–91)	83 (31–99)	82 (45–99)
**BSA [m^2^]**	1.92 ± 0.2 (n = 175)	1.87 ± 0.2 (n = 603)	1.82 ± 0.23 (n = 122)	1.87 ± 0.2 (n = 215)
**BMI** [kg/m^2^]	28.4 (18.7–49.9) (n = 175)	26.4 (16.0–70.3) (n = 603)	25.2 (15.6–45.2) (n = 122)	25.8 (16.7–54.4) (n = 215)
**Renal impairment**				
**<15**	4 (1.7)	59 (8.6)	18 (9.6)	37 (11.5)
**15–29**	29 (12.6)	265 (38.6)	64 (34.2)	177 (55.1)
**30–44**	77 (33.3)	259 (37.8)	62 (33.2)	96 (29.9)
**45–59**	121 (52.4)	103 (15.0)	43 (23.0)	11 (3.4)
[mL/min/1.73 m^2^]				
**No. of drugs**	2636	7202	2387	3558
per patient	11 (4–23)	10 (1–24)	13 (5–22)	11 (2–22)
**No. of drugs with rDRP**	268 ^a^ (10.2)	1275 (17.7)	269 ^a^ (11.3)	367 ^a^ (10.2)
**No. of rDRP**	318	1275	468	393
per patient	1 (1–4)	2 (1–5)	3 (1–13)	1 (1–4)
**Change in prescriptions after renal pharmacists’ interventions**	221 (81.0) (n = 273) ^b^	1090 (85.4)	363 (77.6)	261 (88.2) (n = 296) ^c^

^a^ One drug can have more than one rDRP. ^b^ It was not possible to evaluate the change in prescription when the rDRP ‘Monitoring’ was recommended. ^c^ Documentation of prescription changes started in 07/2020. From this time on, the total number of DRPs were 296.

## Data Availability

The datasets used and/or analyzed during the current study are available from the corresponding author upon reasonable request.

## Abbreviations

The following abbreviations are used in this manuscript:

ADR	Adverse drug reaction
BMI	Body mass index
BSA	Body surface area
CK	Creatine kinase
CKD-EPI	Chronic Kidney Disease Epidemiology Collaboration
CPOE-CDSS	Physician order entry systems with clinical decision support
CPPE	Centre for Pharmacy Postgraduate Education
eGFR	Glomerular filtration rate (estimated)
KKiMK	Catholic Hospital in the Märkisch District
rDRP	Renal-drug-related problems
RI	Renal impairment
RP	Renal pharmacist

## References

[1] Sundström J., Bodegard J., Bollmann A., Vervloet M.G., Mark P.B., Karasik A., Taveira-Gomes T., Botana M., Birkeland K.I., Thuresson M. (2022). Prevalence, outcomes, and cost of chronic kidney disease in a contemporary population of 2·4 million patients from 11 countries: The CaReMe CKD study. Lancet Reg. Health Eur..

[2] Girndt M., Trocchi P., Scheidt-Nave C., Markau S., Stang A. (2016). The Prevalence of Renal Failure. Results from the German Health Interview and Examination Survey for Adults, 2008–2011 (DEGS1). Dtsch. Arztebl. Int..

[3] Trocchi P., Girndt M., Scheidt-Nave C., Markau S., Stang A. (2017). Impact of the estimation equation for GFR on population-based prevalence estimates of kidney dysfunction. BMC Nephrol..

[4] Holm H., Bjerke K., Holst L., Mathiesen L. (2015). Use of renal risk drugs in patients with renal impairment. Int. J. Clin. Pharm..

[5] Seiberth S., Bauer D., Schönermarck U., Mannell H., Stief C., Hasford J., Strobach D. (2020). Correct use of non-indexed eGFR for drug dosing and renal drug-related problems at hospital admission. Eur. J. Clin. Pharmacol..

[6] Seiberth S., Mannell H., Birkenmaier C., Neuerburg C., Smolka V., Andraschko M., Strobach D. (2022). Benefit of medication reviews by renal pharmacists in the setting of a computerized physician order entry system with clinical decision support. J. Clin. Pharm. Ther..

[7] Wolf U., Ghadir H., Drewas L., Neef R. (2023). Underdiagnosed CKD in Geriatric Trauma Patients and Potent Prevention of Renal Impairment from Polypharmacy Risks through Individual Pharmacotherapy Management (IPM-III). J. Clin. Med..

[8] Tesfaye W.H., Castelino R.L., Wimmer B.C., Zaidi S.T.R. (2017). Inappropriate prescribing in chronic kidney disease: A systematic review of prevalence, associated clinical outcomes and impact of interventions. Int. J. Clin. Pract..

[9] Dorks M., Allers K., Schmiemann G., Herget-Rosenthal S., Hoffmann F. (2017). Inappropriate Medication in Non-Hospitalized Patients with Renal Insufficiency: A Systematic Review. J. Am. Geriatr. Soc..

[10] Blix H.S., Viktil K.K., Moger T.A., Reikvam A. (2006). Use of renal risk drugs in hospitalized patients with impaired renal function—An underestimated problem?. Nephrol. Dial. Transplant. Off. Publ. Eur. Dial. Transpl. Assoc.-Eur. Ren. Assoc..

[11] Tuttle K.R., Alicic R.Z., Short R.A., Neumiller J.J., Gates B.J., Daratha K.B., Barbosa-Leiker C., McPherson S.M., Chaytor N.S., Dieter B.P. (2018). Medication Therapy Management after Hospitalization in CKD: A Randomized Clinical Trial. Clin. J. Am. Soc. Nephrol. CJASN.

[12] Pehlivanli A., Eyupoglu S., Basgut B., Erturk S., Ozcelikay A.T. (2023). Impact of a multidisciplinary approach involving clinical pharmacist on resolving drug related problems in chronic kidney patients: A prospective interventional study. BMC Nephrol..

[13] Center for Pharmacy Postgraduate Education Fundamentals of Renal Therapeutics. E-Course. https://www.cppe.ac.uk/programmes/l/renalec-e-01.

[14] Pharmaceutical Care Network Europe Foundation (PCNE) (2020). Classification for Drug Related Problems V9.1. Zuidlaren.

[15] ADKA DokuPIK. https://www.adka-dokupik.de.

[16] Seiberth S., Bauer D., Schönermarck U., Mannell H., Stief C., Hasford J., Strobach D. (2021). Implementation of a renal pharmacist consultant service—Information sharing in paper versus digital form. J. Clin. Pharm. Ther..

[17] Onder G., Petrovic M., Tangiisuran B., Meinardi M.C., Markito-Notenboom W.P., Somers A., Rajkumar C., Bernabei R., van der Cammen T.J. (2010). Development and validation of a score to assess risk of adverse drug reactions among in-hospital patients 65 years or older: The GerontoNet ADR risk score. Arch. Intern. Med..

[18] Guignard B., Bonnabry P., Perrier A., Dayer P., Desmeules J., Samer C.F. (2015). Drug-related problems identification in general internal medicine: The impact and role of the clinical pharmacist and pharmacologist. Eur. J. Intern. Med..

[19] Falconnier A.D., Haefeli W.E., Schoenenberger R.A., Surber C., Martin-Facklam M. (2001). Drug dosage in patients with renal failure optimized by immediate concurrent feedback. J. Gen. Intern. Med..

[20] Meyer-Massetti C., Hofstetter V., Hedinger-Grogg B., Meier C.R., Guglielmo B.J. (2018). Medication-related problems during transfer from hospital to home care: Baseline data from Switzerland. Int. J. Clin. Pharm..

[21] Al Raiisi F., Stewart D., Fernandez-Llimos F., Salgado T.M., Mohamed M.F., Cunningham S. (2019). Clinical pharmacy practice in the care of Chronic Kidney Disease patients: A systematic review. Int. J. Clin. Pharm..

